# Behavioral Effects of Exposure to Phthalates in Female Rodents: Evidence for Endocrine Disruption?

**DOI:** 10.3390/ijms23052559

**Published:** 2022-02-25

**Authors:** Nolwenn Adam, Sakina Mhaouty-Kodja

**Affiliations:** Sorbonne Université, CNRS, INSERM, Neuroscience Paris Seine—Institut de Biologie Paris Seine, 7 quai Saint Bernard, 75005 Paris, France; nolwenn.adam@sorbonne-universite.fr

**Keywords:** behavior, endocrine disruptor, phthalate, sex steroids, reproduction, nervous system

## Abstract

Phthalates have been widely studied for their reprotoxic effects in male rodents and in particular on testosterone production, for which reference doses were established. The female rodent brain can also represent a target for exposure to these environmental endocrine disruptors. Indeed, a large range of behaviors including reproductive behaviors, mood-related behaviors, and learning and memory are regulated by sex steroid hormones. Here we review the experimental studies addressing the effects and mechanisms of phthalate exposure on these behaviors in female rodents, paying particular attention to the experimental conditions (period of exposure, doses, estrous stage of analyses etc.). The objective of this review is to provide a clear picture of the consistent effects that can occur in female rodents and the gaps that still need to be filled in terms of effects and mode(s) of action for a better risk assessment for human health.

## 1. Introduction

Phthalates are among the most abundant organic pollutants in the environment [1], due to their extensive use in the plastic industry. Several phthalates are listed by the European Chemical Agency (ECHA) as substances of very high concern due to their toxicity for reproduction (butyl benzyl phthalate BBP, dibutyl phthalate DBP, dicyclohexyl phthalate DCHP, di(2-ethylhexyl)phthalate DEHP, di-isobutyl phthalate DIBP, diisohexyl phthalate DIHP, di-isopentyl phthalate DIPP, dipentyl phthalate DPP…), and endocrine disrupting properties for human health (BBP, DBP, DCHP, DEHP, DIBP) and the environment (DEHP). Based on their ability to reduce fetal testosterone levels in males, a tolerable daily intake dose of 50 µg/kg/d was initially established by the European Food Safety Authority [2], and this was recently updated and confirmed for BBP, DBP, DEHP, DINP, and DIDP for use in food contact materials [3]. In recent studies, we showed that the adult rodent nervous system is also vulnerable to exposure to DEHP on its own or in a phthalate mixture. The observed effects on reproductive behavior were linked to an endocrine mode of action involving the neural alteration of androgen receptors in males and progesterone receptors in females [4,5]. Interestingly, these effects were observed at doses equivalent to or below the tolerable daily intake (TDI) dose of 50 µg/kg/d in both male and female mice. Moreover, the females appeared more sensitive to phthalate exposure than males since, under comparable experimental conditions, several components of sexual behavior were affected in females [4].

**Figure 1:**
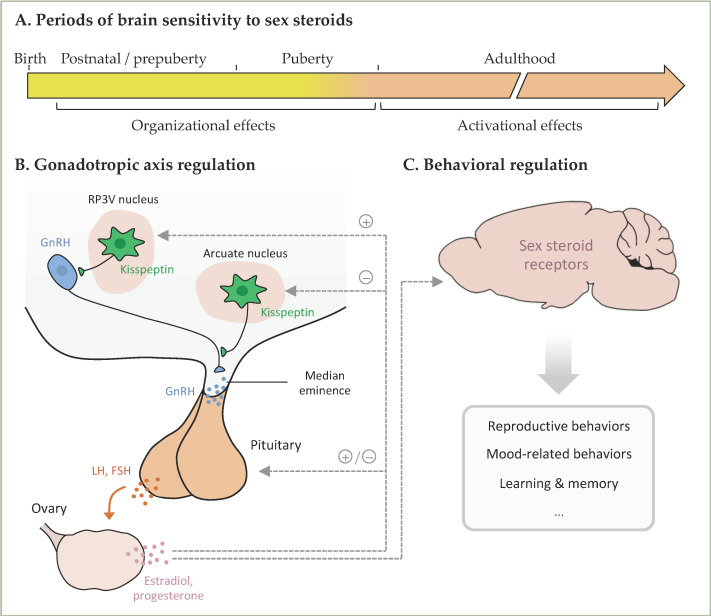
(**A**). In females, the ovaries start synthesizing and liberating sex steroids from postnatal day 7. During the postnatal/prepubertal and pubertal periods, sex steroids play an important role in the organization of neural structures involved in the regulation of the gonadotropic axis and behaviors. During adulthood, sex steroids play an activation role. (**B**). Kisspeptin neurons from the RP3V and arcuate nucleus project on GnRH neurons, thereby stimulating GnRH liberation, which acts on the pituitary to stimulate the liberation of LH and FSH and consequently the ovarian secretion of gonadal hormones. In turn, sex steroids exert positive or negative feedbacks on kisspeptin neurons and the pituitary. (**C**). Ovarian sex steroids act on the brain to activate the neural structures underlying the mentioned behaviors. RP3V, rostral periventricular area of the third ventricle; GnRH, Gonadotropin-Releasing Hormone; LH, Luteinizing Hormone; FSH, Follicle Stimulating Hormone.

Therefore, here we aim to review the experimental studies addressing the behavioral effects and underlying mechanisms of phthalate exposure in female rodents, with a particular focus on behaviors known to be induced or modulated by sex steroid hormones (reproductive and mood-related behaviors and learning and memory). Sex steroid hormones play a key role in the organization of the neural structures that underlie these behaviors during developmental and pubertal periods of life (Figure 1). During adulthood, they are also necessary for the activation of these circuitries, leading to the expression of behavior. The brain undergoes developmental and adult neuroplasticity, regulated by several factors including sex steroid hormones. Changes in hormonal levels or their signaling pathways following exposure to endocrine disrupting compounds (EDCs) can lead to long-term or transient modifications in these processes and consequently in behavior. In this context, previous reviews addressed the effects of exposure to EDCs, including phthalates, on social and reproductive behaviors [6,7,8], or effects of exposure to phthalates on hippocampal plasticity [9] or the nervous system [10]. The present review, in addition to being interested in the effects of female exposure to phthalates on behaviors known to be modulated by sex steroids, aims also to shed light on the experimental conditions used for such studies (phthalate doses, period of exposure, stage of the estrous cycle, etc.). Our ultimate goal is to provide a clear picture of the consistent effects that can occur in female rodents and the gaps that still need to be filled in terms of effects and mode(s) of action for a better risk assessment for human health.

## 2. Hormonal Regulation of Female Behavior

### 2.1. Reproductive Behaviors

Sexual behavior. In rodents, female sexual behavior is restricted to a short period of receptivity that coincides with ovulation. During this period, females emit sexual pheromones, which provide information on their hormonal and receptivity states. During the precopulatory phase, both sexual partners engage in mutual olfactory investigation, which is important in the activation of the following behavioral sequence in both sexes. Once stimulated by female pheromones, the male emits ultrasonic vocalizations and exhibits urinary marking (for review [11]). Thereafter, during the copulatory phase, the male displays several mount episodes with pelvic thrusts and intromissions to which the female responds with a lordosis posture, which consists of having all four paws grounded, the hind region lifted, and the back arched. In mice, copulation ends with male ejaculation, whereas rats only reach satiety after several copulations.

At the neuroanatomical level, male pheromones stimulate the female olfactory bulb, which transmits olfactory information to the medial amygdala and to the bed nucleus of the stria terminalis. These regions send signals to the principal facilitatory system of lordosis behavior, i.e., the ventromedial hypothalamus and to the inhibitory system, which includes the lateral septum, medial preoptic area, and arcuate nuclei. These two systems send projections to the periaqueductal grey regions, which relay information to spinal motoneurons innervating muscles involved in the lordosis posture [12].

These circuitries are tightly regulated by ovarian hormones, estradiol and progesterone. The preovulatory surge of estradiol that occurs during proestrus triggers both the ovulatory surge of LH by the pituitary gland and the upregulation of progesterone receptors (PR) in the neural pathway that underlies female sexual behavior. In rodents, progesterone released after ovarian stimulation by LH acts on neural receptors to induce female receptivity. This hormonal sequence activates the facilitatory system and lifts the inhibition exerted by the inhibitory system to ensure lordosis behavior. Estradiol acts primarily through ERα given that in [13], neural *ERα* deletion greatly reduced the number of PR-immunoreactive neurons in the ventromedial hypothalamus and inhibited lordosis behavior, impaired kisspeptin expression, and arrested cyclicity [13], while in [14], neural *ERβ* deletion had no effect on the adult expression of sexual behavior or regulation of the gonadotropic axis.

Maternal behavior. Female rodents engage in intensive nest building as early as the middle of gestation, with an increased aggression towards intruders. After birth, dams show strong interest in pups and become highly sensitive to olfactory cues from newborns. Nursing behavior is further established by tactile stimulation of the mother by the pups that then gain access to the nipples to suckle. Pups also emit ultrasonic vocalizations; in response, dams exhibit a retrieving behavior to return the pups safely to the nest [15].

In parturient females, olfactory cues integrated by the amygdala are transmitted to the medial preoptic area to promote pup attractivity. The maternal brain is also sensitive to tactile and auditory cues from the pups, which stimulate the bed nucleus of stria terminalis via the cortex. The medial preoptic area and bed nucleus of stria terminalis stimulate other regions, ensuring the establishment and maintenance of maternal behavior, including the ventral tegmental area, nucleus accumbens, and paraventricular nucleus of the hypothalamus [7,16].

The onset of maternal behavior depends on several hormonal events that occur during gestation and parturition. In rodents, estradiol levels are relatively low during the first days of gestation and rise in the last days prior to parturition. Inversely, progesterone levels are high at the beginning and middle of gestation and then drop. The increased ratio of estradiol/progesterone levels at the end of gestation is crucial for uterine preparation for labor. This results in the increased expression of oxytocin and oxytocin receptors, which induce uterine contractions and are also involved in the expression of maternal behavior and milk ejection [17,18]. Among the other hormones involved in maternal behavior, prolactin is secreted through surges by the pituitary at the beginning of gestation and then before parturition. This complex hormonal environment is thus important for maternal responsiveness to pups at parturition. Indeed, the hormonal stimulation of the medial preoptic area and the bed nucleus of stria terminalis is thought to lift the pup avoidance induced by the periaqueductal grey region. Moreover, it activates the dopaminergic system that increases maternal behavior [19]. While the onset of maternal behavior relies on hormones, rodents will progressively become less dependent on hormones and more dependent on sensory and auditory cues from pups that are essential for the maintenance of maternal behavior.

### 2.2. Mood-Related Behaviors

Anxiety- and depressive-like behaviors are also modulated by estrogens, with a diminution of these behaviors during proestrus in both mice and rats [14,20,21,22]. The main brain regions involved in these behaviors are the raphe, amygdala, hippocampus, and prefrontal cortex, with an emerging role of the bed nucleus of stria terminalis [23]. In these structures, the serotonergic system is a strong modulator of mood-related behavior. Cellular bodies of serotoninergic neurons are localized in the raphe nuclei of the brain stem. Tryptophan hydroxylases (TPH) 1 and 2 are the limiting enzymes for serotonin production; TPH2 being the major isoform in neural cells [24]. Serotoninergic neurons send projections to the hippocampus, prefrontal cortex, and amygdala, which all express postsynaptic 5-HT1A receptors [25]. Extracellular levels of serotonin are regulated by serotonin transporters (SERT) that uptake serotonin after its liberation in the synaptic cleft. Dysfunctions in serotonin transmission have been associated with anxiety and depression in both humans [26,27] and rodents [28,29]. In [30,31], estrogens, through ERβ, were shown to mediate the estrogen-mediated modulation of the serotoninergic system, especially by increasing the expression levels of THP2 in the dorsal raphe.

Other neuroendocrine systems are also involved, such as the hypothalamus-pituitary-adrenal (HPA) axis. A constant activation of this axis by chronic stress may lead to its dysregulation and can cause anxiety-related disorders. Activation of the HPA axis in response to stress is modulated by the estrous cycle and estradiol levels [32,33]. Estradiol may also regulate mood-related behaviors through oxytocin and arginine-vasopressin, which were shown to reduce the anxiety- and depression-state levels in [34,35]. In particular, mice deleted for neural *ERβ* display increased anxiety- and depressive-like behaviors that are associated with reduced transcript levels of oxytocin and arginine-vasopressin in the bed nucleus of stria terminalis [20].

### 2.3. Learning and Memory

Learning and memory define the processes by which individuals acquire knowledge, store it, and later retrieve the learned information. Different types of learning and memory processes rely on various synaptic plasticity mechanisms and depend on several brain areas, among which, in [36,37], the hippocampus was shown to play a key role in spatial memory, nonspatial memory such as visual object recognition, and temporal processing of information. Behavioral tests assessing these different aspects of learning and memory are widely used in rodents including in the field of neurotoxicology [38].

Learning and memory are sensitive to gonadal hormones, which exert modulatory effects on these processes throughout the lifespan of the rat. The modulatory effects of estrogens on learning and memory and on underlying processes were extensively studied in females and the underlying mechanisms detailed in previous reviews [39,40,41]. Briefly, in [42], it was shown that estradiol facilitates the establishment of LTP, and in [43,44,45], it was shown that the type and number of dendritic spines are regulated by sex steroid hormones during the estrous cycle. These effects are mediated in part by the classical nuclear ERα and ERβ, given that females globally knocked out for the genes encoding these receptors exhibited impaired spatial learning in [46,47] and reduced hippocampal long-term potentiation (LTP) and plasticity in [48,49]. Non-classical signaling initiated at the cell membrane and inducing rapid modulation of hippocampal plasticity through the activation of second messenger pathways were also described for estradiol [50].

## 3. Behavioral and Neural Effects of Phthalate Exposure

Here we describe the data collected for the effects of exposure to phthalates on reproductive and mood-related behaviors and learning and memory in female rodents. The publications were gathered using the search engine Pubmed, with the keywords “Phthalate and nervous system”, “Phthalate and brain” and “Phthalate and female behavior” up to 30 October 2021.

**Figure 2:**
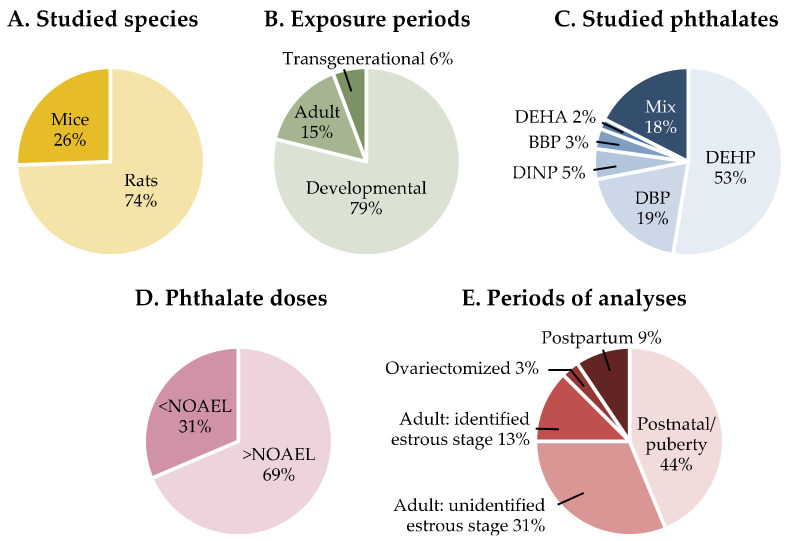
Summary information on the experimental conditions used in the studies discussed in the present review. DEHP, Di-(2-ethylexyl) phthalate; DBP, Dibutyl phthalate; DINP, Diisononyl phthalate; BBP, Benzyl butyl phthalate; DEHA, Di-(2-ethylhexyl) adipate; NOAEL, non-observed adverse effect level.

Information on the gathered articles in terms of studied species, period of exposure, studied phthalates and doses, and age at analyses is presented in Figure 2. The behavioral data are reported in Table 1 for reproductive behaviors, Table 2 for mood behaviors, and Table 3 for learning and memory. Details about the behavioral tests used in the assessment of learning and memory are indicated in Table S3. When neuroanatomical or neuroendocrine observations were made in the same studies, they were reported in the same Table 1, Table 2 and Table 3. Otherwise, in the absence of behavioral analyses, this information can be found in Table S1 for the hypothalamus and Table S2 for brain regions related to cognitive processes. Given the estrogen-mediated regulation of female behavior and neuroendocrine responses, indications were made in the tables with respect to the identification or not of the stage of the estrous cycle in adult females.

### 3.1. Effects of Phthalate Exposure on Reproductive Behaviors

#### 3.1.1. Sexual Behavior

Developmental exposure. Three studies addressed the effects of developmental exposure to high doses of DBP [51,52,53] or DINP [53] on the expression of female sexual behavior in rats (Table 1). One study reported a lower lordosis quotient at all tested doses [53] while the two other studies found no effect [51,52]. These three studies had not only the use of very high phthalate doses in common but also the assessment of lordosis behavior in intact females at the proestrus or estrus stage. Given the great variability in estradiol levels during these two stages, which may then induce differences in the receptivity levels between intact females, sexual behavior is generally measured under normalized hormonal levels through ovariectomy and priming with estradiol and progesterone.

Disruption of sexual behavior can occur either through changes in estrogen levels, progesterone levels, or both or in the expression levels of their receptors. In the study of Lee et al. [53], the behavioral changes were associated with transient reduced hormonal levels at PND7 as no modifications were seen at adulthood as in the study by Guerra et al. [51].

Table S1 shows that in other neuroendocrine and neuroanatomical studies where no behavioral studies were conducted, changes in the levels of LH at 5 or 30 mg/kg/d of DEHP [54,55] and of estradiol at 5 mg/kg/bw of DBP or progesterone at 500 mg/kg/d of DEHP [55,56] were observed in postnatal animals exposed during the prenatal/postnatal or prepubertal/pubertal period. Similarly, changes were seen in neural aromatase activity for exposure to at least 15 µg/kg/d of DEHP [57], or in the expression of ER for exposure to 0.5 mg/kg/d of DBP and of PR at the highest DINP dose tested [56,58]. Modifications in the GnRH/kisspeptin system, which governs the gonadotropic axis, were seen from 5 mg/kg/d for DBP and 1 mg/kg/d of DEHP [56,59]. Whether these changes that impact the developing brain trigger adult reproductive dysfunctions later on requires further studies. Longer estrous cycles were described [55,56] depending on the exposure period [56].

Among the five studies that analyzed the impact of prenatal/postnatal or prepubertal/pubertal exposure in adult females [55,60,61,62,63,64], only two studies identified the stage of the estrous cycle [55,61]. Roepke et al. [61] reported the unchanged expression of *Kiss1*, *ERα*, and *ERβ* in the arcuate nucleus of proestrus females exposed to 500 mg/kg/d of DEHP. Yu et al. [55] confirmed the unaffected *Kiss1* expression in the arcuate nucleus in estrus females exposed to DEHP at 500 or 5 mg/kg/d during the prepubertal/pubertal period, and showed that these two doses induced opposite effects with either higher or lower hypothalamic GnRH expression, *Kiss1* mRNAs levels in the AVPV, and serum levels of LH, estradiol, and progesterone. Altogether, these observations indicate that neuroendocrine changes can occur in the female brain areas that underlie the regulation of the gonadotropic axis following developmental exposure to moderate to low doses of phthalates. Whether these modifications impair the adult expression of sexual behavior still needs further investigations.

Adult exposure. To our knowledge, only one study investigated the effects of chronic adult exposure to phthalates on female sexual behavior [4]. This analysis was performed on ovariectomized females, which were supplemented with estradiol and primed with progesterone before tests to normalize their hormonal levels. It was shown that exposure to low doses of DEHP alone (5 and 50 µg/kg/d) or in an environmental phthalate mixture disrupted both the precopulatory and copulatory phases of sexual behavior. Females exposed to DEHP alone or in a phthalate mixture were less attractive to males, and those exposed to DEHP at 50 µg/kg/d or to the phthalate mixture were unable to discriminate between male and female pheromonal cues. Moreover, the lordosis quotient was diminished while the rejection behavior towards male mounts was increased.

These behavioral alterations were associated with a selective reduced number of PR-immunoreactive neurons in the neural circuitry that underlies sexual behavior (arcuate nucleus, preoptic area, ventromedial hypothalamus, medial amygdala, and bed nucleus of the stria terminalis) [4]. In these regions, no effects were found on the number of ERα-immunoreactive neurons. This suggests that, under normalized hormonal levels, exposure to DEHP alone or in a mixture directly targeted neural structures to lower the expression of female sexual behavior through an endocrine mode of action involving the PR.

Two other studies assessed the effects of acute or sub-chronic exposure to BBP or DEHP in ovariectomized or intact females, respectively [65,66]. Acute exposure of hormonally depleted rats to 10 mg of BBP increased PR expression in the preoptic area only [65], while Liu et al. [66] reported increased hypothalamic GnRH expression and lower hormonal levels following sub-chronic exposure to DEHP at 300 to 3000 mg/kg/d in females at an unidentified estrous stage (Table S1). Apart from the fact that these treatments affected hypothalamic gene expression or hormonal levels, their physiological meaning in the context of gonadotropic axis needs further investigation.

**Table 1:** Effects of phthalate exposure on female reproductive behaviors. Avp, arginine-vasopressin; BBP, benzyl butyl phthalate; Cyp19a1, cytochrome P450 family 19 subfamily member 1; DBP, dibutyl phthalate; DEHA, di-(2-ethylhexyl) adipate; DEHP, Di-(2-ethylexyl) phthalate; DEP, diethyl phthalate; DiBP, diisobutyl phthalate; DINP, diisononyl phthalate; E_2_, estradiol; Esr1 and 2, estrogen receptor 1 and 2; FSH, follicle stimulating hormone; GD, gestational day; GnRH1, Gonadotropin Releasing Hormone 1; Kiss1, kisspeptine 1; LH, luteinizing hormone; Oxt, oxytocin; OVX, ovariectomy; P, progesterone; PND, postnatal day; PNW, postnatal week; PPD, postpartum day; ppm, parts per million.

	Species	Exposure Period	Route	Substances and Doses	Age at Analyses	Behavioral Analyses	Neuroanatomical and Neuroendocrine Analyses/Other Findings	Ref.
Sexual behavior								
Developmental exposure	Wistar rat	F0 adult dams: GD15 to PND21	Oral (diet)	DBP: 20– 200–2000–10,000 ppm DINP: 40–400–4000–20,000 ppm DEHA: 480–2400–12,000 ppm	F1 offspring: Postnatal: PND7 Adult: PNW20-21 at proestrus	• Lordosis behavior ^1^ (PNW20-21, proestrus): lower lordosis quotient at all doses.	• Hypothalamic gene expression PND7: higher granulin expression for DBP-2000, 10,000 and DINP-40, 400, 20,000. Lower p130 expression level for DEHA-480 and 2400. • Hormonal serum levels: lower E_2_ levels for DBP-2000 and DINP-40, but no effect on testosterone levels on PND7. No effect on LH, FSH, and E_2_ levels on PNW20-21 at proestrus.	[53]
	Wistar rat	F0 adult dams: GD12 to PND21	Oral (gavage)	DBP: 100 mg/kg bw/d	F1 offspring: Pubertal: PND30 Adult: PND60-80 at estrus	• Lordosis behavior ^1^ (PND80, estrus): no effect on lordosis quotient: data not shown.	• Pubertal onset (since PND30): no effects on the days of vaginal opening and first estrous. • Estrous cyclicity: no effects on the estrous cycle (PND60-75). • Hormonal levels: no effect on LH, FSH, and P levels (PND75).	[51]
	Sprague Dawleyrat	F0 adult dams: GD14.5 to PND6	Oral (gavage)	DBP: 500 mg/kg bw, every two days	F1 offspring: Postnatal: PND10, 24 and 29 Adult: PND60-90, estrus (for lordosis)	• Lordosis behavior ^1^ (PND60-90, estrus): no effect on lordosis quotient, agonistic (boxing, defensive, biting, frontal, and lateral threat), and kicking behaviors.	• Hypothalamic gene expression PND10, 24 and 90; unidentified estrous stage: increased Esr2 and Gnrh1 and decreased Avp mRNA levels for PND24 only. No effects on *Cyp19a1*, *Esr1*, *Oxt,* and *Kiss1* mRNA levels for all ages. • Pubertal onset (since PND29): no effects on the vaginal opening.	[52]
Adult	C57Bl /6J mouse	Adult: PNW8 to PNW23	Oral (diet)	DEHP: 5–50 µg/kg bw/d Phthalate mix (µg/kg bw/d): DEHP 5, DBP 0.5, BBP 0.5, DiBP 0.5, DEP 0.25	Adult: PNW14-23 ▪ Sexual behavior in OVX and E_2_/P-primed mice ▪ Estrous cyclicity in intact mice	• Lordosis behavior ^1^ (OVX and E_2_/P-primed mice): lower lordosis quotient at DEHP alone or in a mix. • Olfactory discrimination (OVX and E_2_/P-primed mice): loss of preference towards males for DEHP-50 and the phthalates mix. • Attractiveness (OVX and E_2_/P-primed mice): males spent less time investigating cues (awake or anesthetized females, urine) from females exposed to DEHP alone or in a mix. Males emitted less and shorter courtship vocalizations in the presence of females exposed to DEHP alone or in a mix.	• Hormone receptor immunoreactivity (OVX and E_2_/P-primed mice): lower number of PR-immunoreactive cells in the medial amygdala, the ventromedial hypothalamus, and the medial preoptic area for DEHP alone or in a mixture. Lower number of PR-immunoreactive cells in the arcuate nucleus and the bed nucleus of the stria terminalis for DEHP-5. No effects on ER-immunoreactive cells in these regions at tested doses. • Estrous cyclicity: longer estrous cycle: longer estrus and metestrus stages, and shorter proestrus stage.	[4]
Maternal behavior								
Developmental	Sprague Dawley rat	F0 adult dams: GD14 to GD21	Oral (gavage)	DEHP: 10 mg/kg/d	F1 dams: PPD3, 5 and 7 (pup retrieval test) and PPD8	• Pup retrieval test ^1^ (pool of PPD3, 5 and 7): higher first retrieval latency, but lower total retrieval time, total number of retrieved pups, and licking time. No effect on nursing and self-grooming time.	• Oxt (PPD8): lower Oxt levels in the hypothalamus detected by ELISA and Western-Blot and lower plasma levels. Lower mRNA and protein levels of Oxt receptor, with increased gene methylation.	[67]
Adult	Wistar rat	F0 adult dams: GD7 to PND17	Oral (gavage)	DINP: 300–600–750–900 mg/kg bw/d	F0 dams: PND1	• Pup retrieval test ^1^: no effect on the latency to retrieve the pups: data not shown.		[68]
	Long -Evans rat	F0 adult dams: GD0 to PND10	Oral (diet)	Phthalate mix doses ^2^: 200–1000 µg/kg bw/d	F0 dams, PND3-10	• Spontaneous maternal behavior ^1^: no effect on the following behaviors observed during 90 min in their home cage: nursing, licking pups, nest reorganization, time spent outside the nest.		[60]
	C57Bl /6J mouse	F0 adult dams: GD0 to PND10	Oral (diet)	DEHP: 5–40–400 µg/kg bw/d	F0 dams: PND2, 4, 6 F2 dams: PPD2, 4, 6	• Spontaneous maternal behavior ^1^: Behaviors were observed during 30 min in the home cage. F0 dams: no effect on the following behaviors: inside the nest, nursing, licking and grooming pups, digging, eating. F2 dams: no effect on the following behaviors: inside the nest, nursing, licking and grooming pups, digging, eating.		[69]
	C57Bl /6J mouse	F0 adult dams: GD13 to PND16	Oral (diet)	DBP: 50–100 mg/kg/d	F0 dams: PPD4	• Pup retrieval test ^1^: higher latency to retrieve the 2nd pup for all doses, but no effect on the latency to retrieve the 1st and the 3rd pups. • Nest shape analysis: lower nest score for dams of the DBP-50 group.		[70]

^1^ Detailed test protocols are reported in Table S3. ^2^ Composition of the mixture in [60]: 35% DEP, 21% DEHP, 15% DBP, 15% DiNP, 8% DiBP, 5% BBP.

#### 3.1.2. Maternal Behavior

Developmental exposure. One study assessed the effects of perinatal exposure to DEHP at 10 mg/kg/d on pup-retrieving behavior of F1 rats [67]. Exposure increased the latency to retrieve the first pup and lowered the total number of pups retrieved and the time spent licking the pups but did not affect nursing. These behavioral effects were associated with lower hypothalamic levels of oxytocin receptor expression, increased oxytocin receptor methylation, and lower plasmatic oxytocin [67], suggesting that DEHP may reduce maternal behavior by impacting hypothalamic oxytocin signaling.

Adult exposure. Four studies investigated the effects of adult phthalate exposure on maternal behavior, with exposure beginning during gestation and lasting until the lactation period [60,68,69,70]. Lee et al. [70] reported that dams exposed to DBP (50 or 100 mg/kg/d) exhibited a higher latency to retrieve the second pup and created a poor-quality nest in the DBP-50 group. In contrast, no effects of exposure to high doses of DINP (300–900 mg/kg/d) or to a phthalate mixture (0.2 to 1 mg/kg/d) were found on pup retrieval or spontaneous maternal behavior in rats [60,68]. Similarly, exposure to DEHP (5 to 400 µg/kg/d) had no effect on the spontaneous maternal behavior of F0 or F2 mouse dams [69].

### 3.2. Effects of Phthalate Exposure on Mood-Related Behaviors

Developmental exposure. Eight studies (five on rats, three on mice) investigated the effects of prenatal/postnatal or prepubertal/pubertal exposure to DEHP or a phthalate mixture on anxiety-like behavior of F1 offspring, using mainly the elevated plus maze alone or in combination with the open-field (Table 2). Among these studies, seven analyzed these effects on cyclic females [69,71,72,73,74,75,76] and one in postpartum females [67]. Four out of seven studies found increased anxiety-state levels in PND30-35 mice exposed to 5 or 40 µg/kg/d of DEHP [69], in PND42 and adult females at the diestrus or estrus stage exposed to DEHP above 10 mg/kg/d [74,75], and in adult females of unknown estrous stage exposed to DEHP from 1 mg/kg/d [73]. In the study of Xu et al. [75], increased depressive-like behavior was also observed in PND42 and adult females at the diestrus stage. In these studies, increased anxiety-related behavior was associated with impaired HPA axis as evidenced by higher ACTH and lower corticosterone levels together with elevated hypothalamic amounts of GR [73], and lower amounts of hippocampal and striatal ERβ and dopamine receptor 2 [75], with no changes in circulating levels of estradiol [74,75]. The remaining three of seven studies reported no effects in pubertal (PND30, PND42 and PND45) or adult females of unknown estrous stage, which were exposed prenatally or postnatally to DEHP at 30 mg/kg/d [71] or 200 mg/kg/d [76] or to a phthalate mixture at 0.2 or 1 mg/kg/d [72]. Whether this discrepancy is due to differences in the female estrous stage or to other experimental conditions needs further investigation.

No effects of developmental exposure were observed in postpartum females perinatally exposed to 50 or 100 mg/kg/d of DBP [67]. In studies analyzing the transgenerational effects of DEHP exposure in the F3 generation [77,78], no effects were reported in PND30-35 and adult females of unknown estrous stage except at the high dose of 750 mg/kg/d, which seemed to induce a higher time spent in the open arms of the elevated plus maze [77].

**Table 2:** Effects of phthalate exposure on mood-related behaviors. ACTH, adrenocorticotropic hormone; Akt, protein kinase B; AR, androgen receptor; BBP, benzyl butyl phthalate; BDNF, brain-derived neurotrophic factor; CREB, cAMP response element-binding protein; CRH, corticotropin-releasing hormone; D1R and D2R, dopamine receptor 1 and 2; DBP, dibutyl phthalate; DEHP, Di-(2-ethylexyl) phthalate, DEP, diethyl phthalate; DiBP, diisobutyl phthalate; DINP, diisononyl phthalate; DNMT, DNA methyltransferase; E_2_, estradiol; Esr1 and 2, estrogen receptor 1 and 2; ERβ, estrogen receptor β; ERK, extracellular signal-regulated kinases; FSH, follicle stimulating hormone; GD, gestational day; GR, glucocorticoid receptor; LH, luteinizing hormone; MeCP2, methyl-CpG binding protein 2; MR, mineralocorticoid receptor; NR43A1 and 3, Nuclear Receptor Subfamily 4 Group A Member 1 and 3; P, progesterone; PND, postnatal day; PPD, postpartum day.

	Species	Exposure Period	Route	Substance and Doses	Age at Analyses	Behavioral Analyses	Neuroanatomical and Neuroendocrine Analyses/Other Findings	Ref.
Prenatal/postnatal exposure	Long- Evans rat	F0 adult dams: GD2 to PND10	Oral (diet)	Phthalate mix doses ^1^: 200–1000 µg/kg bw/d	F1 offspring: Postnatal: PND25 Adult: PND90, unidentified estrous stage	• Anxiety-like behavior (PND90, unidentified estrous stage): Elevated plus maze: no effect on the time spent and the number of entries in open arms.	• Pubertal onset (since PND25): no effects on the day of vaginal opening.	[72]
	Sprague Dawley rat	F0 adult dams: GD14 to GD21	Oral (gavage)	DEHP: 10 mg/kg/d	F1 dams: PPD8	• Anxiety-like behavior: Elevated plus maze: no effect on the number of open and closed arms entries, proportion of open arms entries, time in the open and closed arms, and percent of the time spent in open arms.	• Hypothalamic BDNF: no effect on BDNF levels. • Plasma BDNF and stress-related hormone levels: higher ACTH levels; no effect on BDNF and corticosterone levels.	[67]
	Wistar rat	PND1 to PND60	Oral (water)	DEHP: 30 mg/kg bw/d	F1 offspring: Postnatal: PND30 Pubertal: PND45 Adult: PND60, unidentified estrous stage	• Anxiety-like behavior: Elevated plus maze: no effect on the frequency of open arms entries and the time spent in open arms, for all ages (PND30, PND45 and PND60 with unidentified estrous stage).		[71]
	Sprague Dawleyrat	PND2 to PND21	Oral (gavage)	DEHP: 10 mg/kg/d	PND57 and PND60, unidentified estrous stage	• Anxiety-like behavior: Open field (PND57): no effect on the number of crossed squares and the number center entries. Elevated plus maze (PND60): lower percentage of open arms entries and time spent in the open arms.	• Hypothalamic protein levels (PND60): higher glucocorticoid receptor level under stressed condition^2^ but not under baseline condition. • Hormonal plasma levels (PND60): higher ACTH levels under baseline and stressed conditions. Lower corticosterone levels under stressed condition, no difference under baseline condition. No effect on P levels.	[73]
	ICR mouse	F0 adult dams: GD7 to PND21	Oral (gavage)	DEHP: 10–50–200 mg/kg/d	F1 offspring: Pubertal: PND42 Adult: PND84, diestrus	• Anxiety-like behavior: PND42: Open field: lower frequency of rearing for DEHP-10 and 200. No effect on the number of grid crossings, the frequency of grooming, and the time spent in the central area. Elevated plus maze: lower number of open arm entries for DEHP-10 and 200. Lower time spent in open arms for all doses. Lower number of total entries for DEHP-10. No effect on the number of unprotected head dips. Dark-light transition: no effect on the time spent and the number of entries in the light chamber. Mirrored maze: no effect on the time spent and the number of entries in the mirrored chamber. PND84 (diestrus): Open field: lower number of grid crossings for DEHP-10 and 200. Higher frequency of grooming for DEHP-10. No effect on the frequency of rearing and the time spent in the central area. Elevated plus maze: lower time spent in open arms for DEHP-50. No effect on the number of open arm entries, the total number of entries and the number of unprotected head dips. Dark-light transition: lower number of entries in the light chamber for all doses. No effect on the time spent in the light chamber. Mirrored maze: lower number of entries in the mirrored chamber for DEHP 10. No effect on the time spent in the mirrored chamber. • Depressive-like behavior: Forced swim test: higher time spent immobile for DEHP-10 for all ages (PND42 and P84 in diestrus)	• Hippocampal protein levels: PND42: lower ERβ level for all doses. Lower p-ERK/ERK ratio for all doses. No effect on AR. PND84 (diestrus): lower ERβ level for all doses. No effect on AR and the p-ERK/ERK ratio. • Hormonal levels (PND42 and 84): no effects on E_2_ serum levels and uterine weight.	[75]
	C57Bl /6J mouse	F0 adult dams: GD0 to PND10	Oral (diet)	DEHP: 5–40–400 µg/kg bw/d	F1 offspring: PND30-35 F3 offspring: PND30-35	• Anxiety-like behavior: Elevated plus maze test F1 offspring: higher time spent in the closed arms for DEHP-5 and 40. No effect on the number of crosses through the center. F3 offspring: no effect on the time spent in the closed arms and the number of crosses through the center.		[69]
	ICR mouse	F0 adult dams: GD6 to GD12; GD13 to GD17	Oral (gavage)	DEHP: 200 mg/kg bw/d	F1 offspring: Pubertal: PND42 Adult: PND56, unidentified estrous stage	• Anxiety-like behavior: PND42: Open field: no effect on the number of fecal particles, the time spent grooming, the latency to enter in the center, and the time spent in the borders, for all periods. Elevated plus maze: no effect on the number of entries and the time spent in open arms, for all gestation periods. PND56 (unidentified estrous stage): Open field: no effect on the number of fecal particles, the time spent grooming, the latency to enter in the center, and the time spent in the borders, for all periods. Elevated plus maze: no effect on the number of entries and the time spent in open arms, for all gestation periods.		[76]
	C57Bl /6J mouse	F0 adult dams: GD7 to GD14	Oral (gavage)	DEHP: 150–200 mg/kg bw/d	F3 offspring: PND35-42	• Anxiety-like behavior: Elevated plus maze: no effect on the time spent in open, middle, and closed sections of the maze.	• Corticosterone serum levels (age at analysis not provided): lower corticosterone level under both baseline and stressed condition ^2^ at DEHP-150 (DEHP-200 not analyzed). • Pituitary gene expression (age at analysis not provided): no effect on the expression of LH and FSH (DEHP-200 not analyzed).	[78]
	CD-1 mouse	F0 adult dams: GD7 to birth	Oral (gavage)	DEHP: 0.02–0.2–500–750 mg/kg/d	F3 offspring: Adult: PND90-120, unidentified estrous stage	• Anxiety-like behavior (PND90-100, unidentified estrous stage): Elevated plus maze: higher time spent in the open arms for DEHP-750. No effect on the number of total arm entries and the number of open arm entries, for all doses.	• Hippocampal gene expression (PND105-120, unidentified estrous stage): no effect on *Esr2*, *GR*, and *DNMT3* expression. • Amygdala gene expression (PND105-120, unidentified estrous stage): down-regulation of *Esr1* for DEHP-200 and 500, *MR* for DEHP-200 and *D2R* for DEHP-20 and 750. No effect on *Esr2*, AR, GR, MR, CRH receptors 1 and 2, melanocortin receptor 4, DNMT1, 3a, 3b, 3l, D1R, and MeCP2 expression.	[77]
Prepubertal/pubertal exposure	Long- Evans rat	PND27 to PND50	Oral (diet)	Phthalates mix doses ^1^: 200–1000 µg/kg bw/d	F1 offspring: Postnatal: PND25 Adult: PND85, unidentified estrous stage	• Anxiety-like behavior (PND85, unidentified estrous stage): Elevated plus maze: higher number of visits to the end of open arms for DEHP-1000. No effect on the time spent and the number of entries in open arms.	• Pubertal onset (since PND25): no effects on the day of vaginal opening.	[72]
	ICR mouse	PND28 to 42	Oral (gavage)	DEHP: 1–10–50–200 mg/kg bw/d	PND84 at diestrus	• Anxiety-like behavior (diestrus): Open field: lower number of grid crossings for DEHP 10 and 50, lower rearing frequency for DEHP 10. Higher frequency of grooming for DEHP 1 and 50. Elevated plus maze: lower number of open arm entries and lower percentage of time spent in open arms, for all doses. No effect on the number of unprotected head dips and total arm entries.	• Striatum protein levels: lower amounts of ERβ for DEHP-1, 10, and 50. Lower amounts of DR2. Lower p-ERK/ERK ratio for DEHP-1 and 10. No effect on the dopamine transporter DAT expression. • Hormonal levels: no effects on E_2_ serum levels and uterine weight.	[74]
Adult exposure	C57Bl /6J mouse	F0 adult dams: GD13 to PPD16	Oral (diet)	DBP: 50–100 mg/kg/d	F0 adult dams: PPD16	• Depressive-like behavior: Forced swim test: mice were placed in a water tank for 6 min, immobility was recorded for 4 min. No effect on immobility time at all doses. Tail suspension test: mice were suspended 50 cm above the floor for 6 min. No effect on immobility time at all tested doses.	*Analyses in the cortex.* • Gene expression (DBP-50 dose only): lower levels for: ABI family member 3 binding protein, activity regulated cytoskeleton associated protein, dual specificity phosphatase 1, early growth response 1, microRNA 212, N-deacetylase and N-sulfotransferase 4, neuromedin B receptor, NR43A 1 and 3, phosphatidylserine decarboxylase, pseudogene 3, T cell receptor alpha variable 9-2, transcription factor AP-2 delta. • Protein expression (DBP-50 dose only): lower levels for: NR43A3, Activity Regulated Cytoskeleton Associated Protein, Early Growth Response 1 and BDNF. Lower phosphorylation of Akt and CREB. • Number of cortical dark neurons (DBP-50 dose only): higher number of Nissl-stained neurons for DBP-50.	[70]

^1^ Composition of the mixture in [72]: 35% DEP, 21% DEHP, 15% DBP, 15% DiNP, 8% DiBP, 5% BBP. ^2^Detailed test protocols are reported in Table S3.

Adult exposure. One study examined the effects of adult exposure to DBP on depressive-like behavior during the lactational period in mice [70]. Exposure of dams to DBP at 50 and 100 mg/kg/d had no effect on the forced swim and tail suspension tests.

### 3.3. Effects of Phthalate Exposure on Learning and Memory and Related Processes

Developmental exposure. Five studies (three on rats, two on mice) analyzed the effects of prenatal/postnatal exposure to DBP, DINP, and DEHP at high doses ranging from 10 mg/kg/d to more than 1 g/kg/d on spatial memory using the Morris water maze [76,79,80,81] or both Morris water and radial arm mazes [68] as shown in Table 3. No treatment effects were reported on the learning of prepubertal and adult female mice [79] or prepubertal or pubertal rats [68,76,80], while performance during the test day seemed to change in three of these studies [68,76,80]. A lower number of neurons in the three hippocampal regions, associated with a higher apoptotic cell death and caspase-3 activity, were observed at PND5 and PND21, but not in adulthood, in rats exposed to DBP at 500 mg/kg/d during the perinatal period [80]. This suggested that the behavioral changes were possibly caused by neurotoxic effects of such high phthalate doses.

Four other studies assessed the effects of exposure to lower doses of phthalates with BBP at 10 µg/kg/d, DEHP at 33 mg/kg/d, or a phthalate mixture at 0.2 to 1 mg/kg/d on aversive stimulus associated learning [82], object recognition memory [83], and cognitive flexibility [72,84], respectively. In mice, the novel object recognition test indicated a higher preference for the novel object in mice exposed to 200 mg/kg/d [83]. Cognitive flexibility tests showed a lower percentage of correct answers and higher percentage of perseveration errors after exposure to a phthalate mixture at 1 mg/kg/d. The cognitive flexibility texts showed a higher percentage of omission errors in rats exposed during the prenatal/postnatal period [84] but not in those exposed during the prepubertal/pubertal period [72]. In particular, the behavioral effects induced by prenatal/postnatal exposure to this phthalate mixture were associated with a lower number of neurons and synapses in the prefrontal cortex and a reduced volume of this brain region in adult females [84]. This phthalate mixture also induced earlier changes given that reduced neurogenesis and increased apoptosis in the cortex of PND5 and PND10 rats at 200–1000 µg/kg/d [85] and the reduced expression of arginine-vasopressin receptor 1B in the prefrontal cortex of PND10 rats at 0.2 mg/kg/d without changing the expression of other signaling pathways on PND10 or adulthood were reported by this laboratory [60].

In the aversive-stimulus associative test, a shorter duration of freezing during the interval period and the tone period was observed in rats exposed to BBP at 10 µg/kg/d [82]. Analyses of the hippocampus and amygdala, the two regions involved in this behavior, showed increased amounts of synapsin-1 in the dorsal hippocampus and lower amounts of ERα and MeCP2 protein in the amygdala [82]. These studies suggest that developmental exposure to even relatively low doses of phthalates can alter morphology and cognitive function related to the hippocampus, cortex, and amygdala.

Table S2 summarizes the data from other studies assessing the effects of developmental exposure to phthalates on brain morphology and key signaling pathways that underlie hippocampal and cortical functions. Three studies assessing the effects of gestational and lactational exposure to DEHP at high doses ranging from 30 to 750 mg/kg/d did not report any effect on hippocampal structural neuroplasticity and protein markers of functional plasticity, or cortical signaling pathways in the hippocampus on PND7, PND14, PND21 [86,87,88], or LTP in adult female rats [86]. Postnatal exposure of rats to 10 mg/kg/d of DEHP affected neither hippocampal neurogenesis nor plasticity in PND26 animals [89,90]. In contrast, modifications were observed in the dopaminergic signaling pathway in the cortex of PND15 mice perinatally exposed to DBP at 50 mg/kg/d [70]. The number of dopaminergic neurons in the substantia nigra and ventral tegmental area of adult females postnatally exposed to 10–20 mg/kg/d of DEHP was also affected [91]. It would have been interesting to perform all these morphological and molecular analyses in parallel in both the developing and adult brain to see whether the reported changes last over adulthood and whether they can explain the behavioral modifications presented above.

**Table 3:** Effects of phthalate exposure on learning and memory. BBP, benzyl butyl phthalate; DBP, dibutyl phthalate; DEHP, di-(2-ethylexyl) phthalate; DEP, diethyl phthalate; DiBP, diisobutyl phthalate; DINP, diisononyl phthalate; E_2_, estradiol; ERα, estrogen receptor α; GD, gestational day; MeCP2, methyl-CpG binding protein 2; mPFC, medial prefrontal cortex; LIMK, LIM domain kinase; NMDA, N-methyl-D-aspartate; NR1 and NR2B, NMDA receptor 1 and 2b; PAK, P21 activated kinase; PND, postnatal day; PPW, postpartum week; ROCK, Rho Associated Coiled-Coil Containing Protein Kinase; Rac, Rac Family Small GTPase; Ube3a, ubiquitin protein ligase E3A.

	Species	Exposure Period	Route	Substance and Doses	Age at Analyses	Behavioral Analyses	Neuroanatomical and Neuroendocrine Analyses/Other Findings	Ref.
Prenatal/postnatal exposure	Sprague Dawley rat	F0 adult dams: GD14 to PND23	Oral (diet)	BBP: 10 µg/mL	F1 offspring: Adult: PND59 and PND65, unidentified estrous stage	• Aversive stimulus associative learning (PND59, unidentified estrous stage): Fear conditioning ^1^*:* shorter duration of freezing during the interval period and during the tone period. No effect on freezing duration during the pretone period	• Hippocampal protein levels (PND65, unidentified estrous stage): increased amounts of synapsin-1 in the dorsal hippocampus, no effect on the ventral hippocampus. No effect on the amounts of MeCP2, Ube3a, and ERα. • Amygdala protein levels (PND65, unidentified estrous stage): lower MeCP2 and ERα levels. No effect on the amounts of Ube3a and synapsin-1. • Hormonal levels (PND65, unidentified estrous stage): no effects on E_2_ serum levels (data not shown).	[82]
	Sprague Dawley rat	F0 adult dams: GD6 to PND21	Oral (gavage)	DBP: 500 mg/kg bw/d	F1 offspring: Postnatal: PND5, 21, 22	• Spatial learning and memory (PND22): Morris water maze ^1^: no effect on the learning period. Lower number of platform crossings during the test session.	• Number of neurons in the hippocampus: lower number of Nissl-stained cells in the dentate gyrus, CA1 and CA3 at PND5 and 21. • Apoptotic cell-death in the hippocampus: higher number of TUNEL-positive cells at PND5 and 21. Higher percentage of apoptotic cells at PND5 and 21 (measured by flow cytometry). • Caspase-3 activity and synaptophysin amount in the hippocampus: higher caspase-3 (involved in apoptosis) activity at PND5 and 21. Lower amounts of synaptophysin protein at PND21 (not analyzed for PND5).	[80]
	Wistar rat	F0 adult dams: GD6 to PND28	Oral (diet)	DBP: 0.037–0.111–0.333–1% of diet (30–55 to 797–1483 mg/kg)	F1 offspring: Pubertal: PND28 and PND35	• Spatial learning and memory (PND35): Morris water maze ^1^: no effect on the test session and visible platform session.	• Weight (PND28): no effect on brain and uterine weights.	[81]
	Wistar rat	F0 adult dams: GD7 to PND17	Oral (gavage)	DINP: 300, 600, 750, 900 mg/kg bw/d	F1 offspring: Adult: PND90 in estrus, and PNW8 to PNW20 in unidentified estrous stage	• Spatial learning and memory: Radial arm maze (PNW20, unidentified estrous stage): no effect on performance (data not shown). Morris water maze ^1^ (PNW8, unidentified estrous stage): no effect on learning performance. Lower swim distance and shorter latency to reach the platform the 1st day at DINP-900. No effect on the 2nd day and when the platform was moved to a new position (data not shown).	• Weight analysis (PND90, in estrus): no effect on uterine, ovarian, and body weight.	[68]
	Long-Evans rat	F0 adult dams: GD2 to PND10	Oral (diet)	Phthalate mix doses ^2^: 200–1000 µg/kg bw/d	F1 offspring: Adult: PND85-90 and PND103-134, unidentified estrous stage	• Cognitive flexibility (PND 85-90, unidentified estrous stage): Attentional set-shift test ^1^: lower percentage of correct answers at all doses. Higher percentage of perseveration errors for Mix-1000, and higher percentage of omission errors for all doses.	• Number of neurons and glia in the mPFC (PND103-PND134, unidentified estrous stage): lower number of neurons at all doses. No effect on glial number. • Synapses in the mPFC (PND103-PND134, unidentified estrous stage): lower number of synaptophysin boutons at all doses. No effect of the number of synapses per neuron. • Volume of the mPFC (PND103-PND134, unidentified estrous stage): lower mPFC volume for all doses. No effect on the white matter volume.	[84]
	C57Bl /6J mouse	F0 adult dams: exposed 2 weeks before mating, to PND21	Oral (diet)	DEHP: 33 mg/kg bw/d	F1 offspring: Adult: PNW37 to PNW39, unidentified estrous stage	• Recognition memory (PNW37 to PNW39, unidentified estrous stage: Novel object recognition ^1^: increased ratio of the time spent investigating the novel object versus the familiar object, indicating a higher preference towards the novel object.		[83]
	ICR mouse	F0 adult dams: GD6 to GD12 and GD13to GD17	Oral (gavage)	DEHP: 200 mg/kg bw/d	F1 offspring: Age at analysis not clear, estrous stage not identified.	• Spatial learning and memory: Morris water maze ^1^: no effect on the learning period for all exposure periods. Higher latency to enter the platform area and shorter time spent in the area during the test session for mice exposed from GD6-GD12 but not GD13-GD17. No effect on the of path length in the platform area and the number of platform crossings for all exposure periods.		[76]
	ICR mouse	F0 adult dams: GD7 to PND21	Oral (gavage)	DEHP: 10–50–200 mg/kg/d	F1 offspring: Pubertal: PND42 Adult: PND84, diestrus	• Spatial learning and memory (PND42 and 84, diestrus): Morris water maze ^1^: no effect on learning and percentage of time in quadrant during the test trail at the two tested ages.	• Hippocampal protein levels (PND42 and 84, diestrus): no effect on the amounts of NMDA receptors NR1 and NR2B. • Hormonal levels (PND42 and 84, diestrus): no effects on E_2_ serum levels and the ratio uterine/body weight.	[79]
Pubertal	Long -Evans rat	PND27 to PND50	Oral (diet)	Phthalate mix doses ^2^: 200–1000 µg/kg bw/d	Postnatal: PND25 Adult: PND87-99, unidentified estrous stage	• Cognitive flexibility (PND87-99, unidentified estrous stage): Attentional set-shift test ^1^: no effect on the percentage of correct answer and perseveration errors for all doses.	• Pubertal onset (since PND25): no effects on the day of vaginal opening.	[72]
Adult	ICR mouse	Adult, for 5 weeks. Age of beginningof treatment is unclear	Oral (gavage)	DBP: 10–50–250 mg/kg bw/d	Adult, after 5 weeks of treatment, unidentified estrous cycle	• Spatial learning and memory (adult, unidentified estrous stage): Morris water maze ^1^: no effect on the path length nor the time spent in quadrant during the test day. No effect on the number of platform crossings nor the swimming speed.	• Hippocampal protein levels (adult, unidentified estrous stage): lower p-LIMK1/LIMK ratio for all doses. Lower amounts of p-PAK1/PAK1 ratio and GTP-Rac1 at DBP-50 and 250. Higher p-cofilin/cofilin ratio and GTP-RhoA level at DBP-50 and 250. Higher p-LIMK2/LIMK ratio at DBP-250. No effect on ROCK level.	[92]

^1^ Detailed test protocols are reported in Table S3. ^2^ Composition of the mixture in [72,84]: 35% DEP, 21% DEHP, 15% DBP, 15% DiNP, 8% DiBP, 5% BBP.

Adult exposure. A study performed in ICR mice showed that sub-chronic exposure to DBP at doses ranging from 10 to 250 mg/kg/d did not affect spatial memory measured in the Morris water maze, despite molecular changes reported in the hippocampus [92].

## 4. Discussion

Beyond the reduced testosterone production reported for phthalate exposure and used to establish the reference doses for these molecules, the female brain can also represent a sensitive target for these environmental chemicals. It appears, from the present review, that the exposure of rodents to phthalates, in particular at doses in the range of or lower than the no-observed adverse effect level (NOAEL of 5 mg/kg bw/d) or TDI doses may affect reproductive and mood-related behaviors and learning and memory. In some cases, behavioral modifications were associated with an endocrine mode of action involving alterations in neural PR immunoreactivity [4], the amounts of hippocampal and striatal ERβ [75], the amounts of ERα protein in the amygdala [82], oxytocin signaling [65], HPA axis [73], or the expression of arginine-vasopressin receptor 1B in the prefrontal cortex [60]. However, several aspects still need further attention to achieve a better view on the potential effects of environmental exposure to phthalates on female brain and behavior and underlying endocrine mode(s) of action. First, in the experimental studies addressing the female behaviors presented in this review, the endocrine mode(s) of action that underlie the observed effects are not systematically characterized. Indeed, while measurement of circulating levels of sex steroids and brain expression of their receptors is generally performed in parallel in the studies assessing reproductive behaviors, this is not always the case for the other behaviors. Only 50% of the total studies analyzing mood-related behavior and 20% of those analyzing learning and memory addressed this aspect. Second, a majority of available studies used phthalate doses in the mg to g range, higher than the NOAEL established for these substances (Figure 2). This could be barely relevant for the doses estimated for the environmental exposure to phthalates, which are in general at the microgram or nanogram range [93]. Moreover, the endocrine mode(s) of action characterized for an adverse effect induced by high doses of a substance can be secondary to other general effects; this point is not specific to phthalates or females but concerns many other substances and both sexes. Third, several analyses of pubertal or adult females did not report the stage of the estrous cycle (Figure 2). This greatly hampers the correct interpretation of data given the modulation of several reproductive and non-reproductive behaviors by sex steroids. In this context, it is important to mention that it is not necessary to use four groups of females with each corresponding to a stage of the estrous cycle. The estrous cycle can be monitored in cyclic females during the period of analyses, and females can be divided into two groups with one corresponding to the estrus-proestrus phase (high estrogen levels) and the other one to the diestrus-estrus stage (low estrogen levels) as previously reported for analyses of the anxiety- or depressive-related behaviors [14,20]. Finally, adulthood is still an underestimated period of exposure with only a few studies analyzing the effects induced by phthalate exposure in adult females (Figure 2). Previous studies showed that the adult brain can also be impacted by endocrine disruptors including phthalates in both sexes [4,5,94,95,96,97,98]. Given the critical role of several brain regions in the regulation of female behaviors and the potential transgenerational effects of many chemical substances including phthalates on descendants, adulthood should be studied as much as the developmental periods.

In humans, epidemiological studies reported associations between exposure to phthalates and low interest in sexual activity in women [99]. Previous systematic reviews of the literature also support an association between prenatal exposure to phthalates and adverse cognitive outcomes and neurological disorders [100,101]. Other studies described an association between urinary phthalates and anxiety or depressive symptoms in young adults [102] or the elderly population [103]. It is possible that these effects in humans occur through disruption of endocrine systems including those related to sex steroids. Indeed, it is largely known that sex steroids are not only mandatory for sexual function but that they also influence anxiety state level and cognition [104,105,106]. Therefore, the adverse effects and mode(s) of action reported in rodents following environmental exposure to phthalates might be also relevant for humans.

## Supplementary Materials

The following supporting information can be downloaded at: https://www.mdpi.com/article/10.3390/ijms23052559/s1.
