# Supplementary material for: Behavioral Effects of Exposure to Phthalates in Female Rodents: Evidence for Endocrine Disruption?
Source: Int J Mol Sci. 2022 Feb 25;23(5):2559. doi: 10.3390/ijms23052559 (PMC8910129; doi:10.3390/ijms23052559)
Supplement: Supplementary file 1 [file ijms-23-02559-s001.zip › ijms-1597747-supplementary.pdf]

Supplemental Material

Behavioral effects of exposure to phthalates in female rodents: evidence for endocrine disruption?

Nolwenn Adam and Sakina Mhaouty-Kodja

Table of Contents

Table S1. Neuroanatomical and neuroendocrine effects of phthalate exposure on the female hypothalamus.

Table S2. Neuroanatomical and neuroendocrine effects of phthalate exposure on brain regions related to cognitive processes.

Table S3. Details on the behavioral tests used.

Table S1. Neuroanatomical and neuroendocrine effects of phthalate exposure on the female hypothalamus. Akt, protein kinase B; Avp, arginine-vasopressin; Avp1ra and 1b, arginine-vasopressin receptor 1a and 1b; AVPV, Anteroventral periventricular nucleus; BBP, benzyl butyl phthalate; BDNF, brain-derived neurotropic factor; DBP, dibutyl phthalate; DEHP, Di-(2-ethylexyl) phthalate, DEP, diethyl phthalate; DiBP, diisobutyl phthalate; DINP, diisononyl phthalate; E_2, estradiol; ER α and β, estrogen receptor α and β; Esr1 and 2, estrogen receptor 1 and 2; FSH, follicle stimulating hormone; GABA-B, gamma-aminobutyric acid receptor B; GD, gestational day; GnRH, Gonadotropin Releasing Hormone; GnRH , GnRHR receptor; GPR54, kisspeptin receptor; IGF-1, insulin-like growth factor 1; LD, lactational day; LH, luteinizing hormone; mTOR, mammalian target of rapamycin; Oxt, oxytocin; OxtR, oxytocin receptor; OVX, ovariectomy; P, progesterone; PND, postnatal day; PNW, postnatal week; ppm, parts per million; PR, progesterone receptor; TH, tyrosine hydroxylase.

	Specie	Exposure period	Route	Substance and doses	Age at analyses	Neuroanatomical & neuroendocrine analyses	Other findings	Ref
Prenatal / postnatal	Long-Evans rat	F0 adult dams: GD0 to PND10	Oral (diet)	Phthalate mix doses[1]: 200 - 1000 μg/kg bw/d	F1 offspring: Postnatal: PND10 and PND30 Adult: PND90, unidentified estrous stage	• Hypothalamic gene expression:<br>PND10: no effect on *Avp*, *Avpr1a* and *Avpr1b*, Oxt and Oxtr expression.<br>PND90 (unidentified estrous stage): no effect on *Avp*, *Avpr1a* and *Avpr1b*, *Oxt* and *Oxtr* expression.	• Pubertal onset (since PND30): no effects on the day of vaginal opening	[60]
	Wistar rat	F0 adult dams: GD1 to PND15	Oral (drinking water)	DEHP: 3 - 30 mg/kg bw/d	F1 offspring: PND15	• Hypothalamic neurotransmitter: higher aspartate and GABA concentration for DEHP30, no effect on glutamate concentration.<br>• Hormonal levels: higher LH serum level at DEHP-30. No effect on FSH serum level.<br>• Reproductive organ weight: no effects on uterine or ovarian weights.		[54]
	Wistar r	F0 adult dams: GD6 to LD21	Oral (gavage)	DEHP: 0.015 - 0.045 - 0.135 - 0.405 - 1.215 - 5 - 15 - 45 - 135 - 405 mg /kg bw/d	F1 offspring: Postnatal: PND1, PND22	• Aromatase activity in the preoptic area:<br>PND1: no effects on the aromatase activity.<br>PND22: increased aromatase activity for all doses, except for DEHP-0.045 and 5.		[57]
	Fischer CDF rat	F0 adult dams: GD11 to PND7	Dams: I.p. injection Pups: sc injection	DEHP: 500 mg/kg/d	F1 offspring: Postnatal: PND28 Adult: PND70-90 at proestrus	• Gene expression in the arcuate nucleus (proestrus): lower adiponectin receptor 1 gene expression level. No effect on the expression of ERα, ERβ, kisspeptin-1, BDNF, cannabinoid receptor 1, GABA-B receptor 1 and 2, melanocortin 3 receptor, neuropeptide Y, NPY Y2 receptor, opioid receptor and TH.	• Pubertal onset (since PND 28): delayed vaginal opening.	[61]
	Sprague-Dawley rat	F0 adult dams: GD14 to birth	Oral (gavage)	DEHP: 1 - 20 - 50 - 300 mg/kg bw/d	F1 offspring: Adult: PND60-68, unidentified estrous stage	• Hypothalamic gene expression (unidentified estrous stage): no effect on *ERα*, *ERβ*, *PR*, GnRH, *Kiss1* and *Kiss1R* gene expression.<br>• Hormonal serum levels: lower E_2 levels in proestrus for DEHP-300, but no change for other estrous cycle stages. No effect on P levels for all estrous cycle stages. Higher FSH levels in proestrus for DEHP-1, 50 and 300 and higher FSH levels in estrus for DEHP-50 and 300, but no change for other estrous stages.	• Ovarian gene expression: no effect on FSH and LH receptors gene expression.<br>• Pituitary gene expression: no effect on Esr1, Esr2, PR, GnRHR, FSH and LH gene expression.	[62]
	Sprague-Dawley rat	PND1 to PND5	Sc injection	DBP: 0.5 - 5 - 50 mg/kg bw/d	PND25 and after the two first estrous cycles (in proestrus)	• Gene expression in the AVPV (proestrus): higher kisspeptin and GPR54 expression at DBP-5. Higher ERβ expression at DBP-50. No effect on the ERα expression.<br>• Gene expression in the arcuate nucleus (proestrus): higher kisspeptin expression at DBP-5, and lower GPR54 expression at all doses. Lower ERα expression at DBP-50, and ERβ expression at DBP-0.5.<br>• Kisspeptin immunoreactivity (proestrus): higher kisspeptin immunoreactivity density in the AVPV at DBP-0.5 and in the arcuate nucleus at DBP-5.<br>• Hormonal serum levels (proestrus): higher E_2 level at DBP-5, but no effect on LH level.	• Pubertal onset (since PND25): advanced vaginal opening.<br>• Estrous cyclicity: no effect on the estrous cycle duration.<br>• Ovarian weight (proestrus): higher ovary/body weight ratio at DBP-5 and 50.	[56]

	CD (SD)IGS rat	GD15 to PND10	Oral (diet)	DINP: 4000 - 20000 ppm	PND10	• Gene expression in the medial preoptic area: lower PR expression for DINP-20000, but no effect on ERα, ERβ, and steroid receptor coactivator-1 (SRC-1) gene expression.		[58]
Prepubertal /pubertal exposure	Wistar rat	PND15 to PND38	Oral (gavage)	DEHP: 0.2 - 1 - 5 mg/kg bw/d	PND38, at first estrus	• Gene expression and protein levels in the hypothalamus: higher GnRH mRNA levels for DEHP-1 and 5, and higher protein levels at all doses. Higher IGF-1 mRNA levels for DEHP-1 and 5, higher protein levels for all doses and higher IGF-1 concentration at DEHP-5. Higher IGF-1R mRNA levels for DEHP-5 and higher protein levels at all doses. Higher mTOR mRNA and protein levels at DEHP-1 and 5. Higher Akt MRA levels for DEHP-5 and higher protein expression at DEHP-0.2 and 5. Higher PI3K protein levels at all doses, but no effect on the mRNA levels.<br>• Neurons number and apoptosis: higher number of Nissl-stained cells for DEHP-5. Lower apoptosis rate (number of TUNEL-positive cells) for all groups.<br>• Hormonal levels: higher IGF-1 and GnRH serum levels at DEHP-1 and 5.	• Pubertal onset: advanced vaginal opening for DEHP-5.	[59]
	Wistar rat	PND22 to PND50	Oral (gavage)	DEHP: 250 - 500 - 1000 mg/kg bw/d	PND50, unidentified estrous stage	• Hypothalamic GnRH content (unidentified estrous stage): higher GnRH level for DEHP-500 and 1000 (quantified by ELISA).<br>• Hypothalamic GnRH expression (unidentified estrous stage): no effect on the GnRH gene expression in the hypothalamus but higher GnRH protein levels for all doses.<br>• Hypothalamic GnRH immunoreactivity (unidentified estrous stage): higher GnRH immunoreactivity mean density and integral density for all doses.	• Uterine GnRHR analysis (unidentified estrous stage): higher GnRHR gene expression in the uterus for DEHP-1000. Higher GnRHR protein level in the uterus for DEHP-1000 and lower for DEHP-250 and 500. Higher GnRHR immunoreactivity mean density and integral density for all doses.	[63]
	Wistar rat	PND22 to PND50	Oral (gavage)	DEHP: 250 - 500 - 1000 mg/kg bw/d	PND50, unidentified estrous stage	• IGF-1 and GnRH levels in the hypothalamus (unidentified estrous stage): higher IGF-1 levels for DEHP-1000 and lower IGF-1 levels for DEHP-250 and 500. Higher GnRH levels for DEHP-500 and 1000. IGF-1 and (GnRH quantified by ELISA).<br>• Hypothalamic gene expression (unidentified estrous stage): lower kisspeptin expression for all doses. Higher GPR54 expression for DEHP 500. No effect on GnRH expression.<br>• Hypothalamic protein immunoreactivity (unidentified estrous stage): higher kisspeptin and GnRH immunoreactivity integral density for all doses. Higher GPR54 immunoreactivity integral density for DEHP-500.<br>• Hypothalamic protein levels (unidentified estrous stage): higher kisspeptin and GnRH protein levels for all doses. Higher GPR54 protein levels for DEHP-500 and 1000.<br>• Hormonal serum levels (unidentified estrous stage): higher GnRH levels for all doses. Lower FSH, LH and testosterone, and higher P levels for DEHP-500 and 1000. No effect on the IGF-1 and E_2 levels.	• Pubertal onset: delayed vaginal opening for DEHP-1000.<br>• Estrous cyclicity: longer estrous cycle for DEHP-500 and 1000, with a longer anestrus phase.	[64]

	Sprague-Dawley rat	PND22 to PND70	Oral (gavage)	DEHP: 5 - 500 mg/kg bw/d	PND22, PND28 and PND70-73 (in estrus)	• GnRH expression in the whole hypothalamus: higher GnRH mRNA levels at DEHP-5 at all ages, but lower levels at DEHP-500.<br>• Kisspeptin expression in the AVPV and the arcuate nucleus: higher kisspeptin mRNA levels at DEHP-5 in the AVPV at all ages, but lower levels at DEHP-500. No effect on kisspeptin mRNA levels in the arcuate nucleus for all doses and all ages.<br>• Hormonal serum levels (PND28 and 70-73): higher LH, E2 and P level at DEHP-5 at all ages, but lower LH, E2 and P levels at DEHP-500. No effect on FSH level, at all ages and doses.	• Pubertal onset (since PND22): advanced vaginal opening at DEHP-5 but delayed at DEHP-500.<br>• Estrous cyclicity: longer estrous cycle at DEHP-500, with longer proestrus and estrus stages.<br>• Ovary and morphology (PND70-73): lower ovarian weight and ovary/body weight ratio at DEHP-500. Lower number of corpora lutea in ovaries at DEHP-500.	[55]
	Sprague-Dawley rat	PND26 to PND30	Sc injection	DBP: 0.5 - 5 - 50 mg/kg bw/d	PND25 and after the two first estrous cycles (in proestrus)	• Gene expression in the AVPV (proestrus): higher kisspeptin expression at DBP-0.5 and lower at DBP-5. Higher GPR54 expression at DBP-0.5 and lower at DBP-50. Higher ERα expression at all doses., no effect on the ERβ.<br>• Gene expression in the arcuate nucleus (proestrus): lower kisspeptin expression at DBP-5 and 50. Higher GPR54 expression at DBP-0.5 and 50 and lower at DBP 5. No effect on the ERα and ERβ expression.<br>• Kisspeptin immunoreactivity (proestrus): no effect on the kisspeptin immunoreactivity density in the AVPV. Lower kisspeptin immunoreactivity density for all doses in the arcuate nucleus.<br>• Hormonal serum levels (proestrus): higher E_2 level at DBP-0.5, and no effect on LH levels.	• Pubertal onset (since PND25): advanced vaginal opening.<br>• Estrous cyclicity: longer estrous cycle at DBP-0.5 and 50.<br>• Ovarian weight (proestrus): higher ovary/body weight ratio at DBP-5 and 50.	[56]
Adult exposure	Wistar rat	PNW8	Sc injection of OVX females.	BBP: 10 mg, one injection	24h after injection.	• Gene expression the preoptic area (POA) and the medial basal hypothalamus (MBH) (OVX females): higher PR mRNA levels in the POA, but no effect in the MBH. No effect on mRNA levels of neurotensin in the POA and on preproenkephalin in the MBH.		[65]
	Wistar rat	PNW9 to PNW13	Oral (gavage)	DEHP: 300 - 1000 - 3000 mg/kg/d	PNW 13, unidentified estrous stage	• Hypothalamic GnRH analysis (unidentified estrous stage): higher GnRH levels for DEHP-1000 and 3000 (quantified by ELISA). Higher GnRH protein level in the hypothalamus for all doses, but no effect on gene expression. Higher GnRH immunoreactivity mean and integral density for all doses.<br>• Hormonal serum levels (unidentified estrous stage): lower P, E_2 and LH levels for DEHP-1000 and 3000. Lower testosterone and FSH levels for DEHP-3000.	• GnRHR expression in the pituitary (unidentified estrous stage): higher GnRHR gene expression in the pituitary for all doses. Higher GnRHR immunoreactivity mean density for DEHP-1000 and 3000. Higher GnRHR immunoreactivity for DEHP-3000.	[66]

[1] Composition of the mixture in [60]: 35% DEP, 21% DEHP, 15% DBP, 15% DiNP, 8% DiBP, 5% BBP.

Table S2. Neuroanatomical and neuroendocrine effects of phthalate exposure on brain regions related to cognitive processes. AR, androgen receptor; AVP1 RA and 1B, arginine-vasopressin receptor 1A and 1B; BBP, benzyl butyl phthalate; BDNF, brain-derived neurotropic factor; D1R and D2R, dopamine receptor 1 and 2; DBP, dibutyl phthalate; DEHP, Di-(2-ethylexyl) phthalate, DEP, diethyl phthalate; DiBP, diisobutyl phthalate; DINP, diisononyl phthalate; ER α and β, estrogen receptor α and β; ERK, extracellular signal-regulated kinases; GD, gestational day; JNK1, mitogen-activated protein kinase 8; LIMK, LIM domain kinase; MAP2, micro-tubule-associated protein 2; Nurr1, nuclear receptor subfamily 4 group A member 2; OTR, oxytocin receptor; PND, postnatal day; PDE 10a, phosphodiesterase 10a; Pitx3, paired like homeodomain 3; TH, tyrosine hydroxylase; THRα, thyroid hormone receptor α.

	Specie	Exposure period	Route	Substance and doses	Age at analyses	Neuroanatomical & neuroendocrine analyses / other findings	Ref
Prenatal / postnatal exposure	Sprague-Dawley rat	F0 adult dams: GD6 to PND21	Oral (gavage)	DBP: 500 mg/kg bw/d	F1 offspring: Adult: PND60, unidentified estrous stage	• Number of neurons in the hippocampus: no effect on the number of Nissl-stained cells in the dentate gyrus, CA1 and CA3.<br>• Apoptotic cell-death in the hippocampus (PND60, undetermined estrous stage): no effect on the number of TUNEL-positive cells and the percentage of apoptotic cells (measured by flow cytometry).<br>• Caspase-3 activity and synaptophysin expression in the hippocampus: no effect on the caspase-3 (enzyme involved in apoptosis) activity and the synaptophysin expression level.	[80]
	Wistar rat	F0 adult dams: GD0 to PND21	Oral (gavage)	DEHP: 30 - 300 - 750 mg/kg bw/d	F1 offspring: Postnatal: PND7, PND14 and PND21	• Pyramidal neurons in the CA1 region of the hippocampus: no effects on the total dendritic length, total number of branching points and number of dendritic intersections for basal and apical dendrites at all ages.<br>• Hippocampal protein levels: no effects at all ages and doses on levels of MAP2c, p-MAP2c, stathmin, p-stathmin, JNK1 and p-JNK1.	[88]
	Wistar rat	F0 adult dams: GD0 to PND21	Oral (gavage)	DEHP: 30 - 300 - 750 mg/kg bw/d	F1 offspring: Postnatal: PND7, PND14 and PND21 Adult: PND80, unidentified estrous stage	• Ultrastructural plasticity in hippocampus (PND7, PND14 and PND21): no effect on the width of the synaptic cleft and the thickness of the post-synaptic density for all doses and all ages. No effect on the density and the morphology of the dendritic spines of pyramidal cells from the CA1 region, for all doses and all ages.<br>• Protein levels in the hippocampus (PND7, PND14 and PND21): no effect at all ages and doses on synaptophysin, PSD-95, G-actin, F-actin, drebrin, PAK 1/2/3 and p-PAK 1/2/3, LIMK-1 and p-LIMK-1, cofilin and p-cofilin, total Rac-1 and GTP-Rac1.<br>• LTP induction (PND80, unidentified estrous stage): no effect on the LTP induction for all doses.	[86]
	Wistar rat	F0 adult dams: GD0 to PND21	Oral (gavage)	DEHP: 30 - 300 - 750 mg/kg bw/d	F1 offspring: Postnatal: PND7	• Protein levels in the cortex: no effect on levels of the following proteins: BDNF, ERK 1/2 and p-ERK 1/2, c-fos and p300.	[87]
	Long-Evans rat	PND16 -22	I.p. injection	DEHP: 10 mg/kg/d	PND26	• Immunofluorescent labeling in the hippocampus: no effect on the number of doublecortin+ and calbindin+ neurons in the dentate gyrus. No effect on the number of synaptophysin-expressing cells in the CA3.<br>• Number of neurons: coloration with Cresyl Violet: no effect on the number of cell bodies in dentate gyrus, CA1 and CA3 regions.	[89]
	Long-Evans rat	PND16 -22	I.p. injection	DEHP: 10 mg/kg/d	PND26	• Spine density of hippocampal cells: no effects on the density of apical and basal dendrites of dorsal CA3 neurons, dorsal CA1 neurons and dorsal dentate gyrus cells.<br>• Hippocampal gene expression: no effects on the BDNF and the caspase-3 mRNA levels in the dorsal hippocampus.	[90]
	Long-Evans rat	PND16 -22	I.p. injection	DEHP: 1 - 10 - 20 mg/kg/d	PND78, unidentified estrous stage	• Tyrosine-hydroxylase (TH) synthesis in the brain: lower TH+ neurons/cresyl-violet stained cells at DEHP-20 in the substantia nigra (SN).): Higher TH+ neurons/cresyl-violet stained cells at DEHP-10 int the ventral tegmental area (VTA)<br>• Gene expression in the SN/VTA region: Higher *Th* and *Pitx3* mRNA exposure at all doses. Higher *Nurr1* mRNA expression.	[91]
	Long-Evans rat	F0 adult dams: GD0 to PND10	Oral (diet	Phthalate mix doses[1]: 200 – 1000 μg/kg bw/d	F1 offspring: Postnatal: PND10 Adult: PND90, unidentified estrous stage	• Gene expression in the mPFC (PND 10):<br>PND10: mRNA levels of AVPr1B were reduced for Mix 200. No effect on LDL receptor, cypP450 11A1, steroidogenic acute regulatory protein, AR, cyp450 19a1, ERα and β, Estrogen-related receptor γ, CD38, OTR, AVPr1A, THRα and D1R and D2R.<br>PND90 (unidentified estrous stage): No effect on LDL receptor, cypP450 11A1, steroidogenic acute regulatory protein, AR, cyp450 19a1, ERα and β, estrogen-related receptor γ, CD38, OTR, AVPr1A and 1B, THRα and D1 and D2R.	[60]
	Long-Evans rat	F0 adult dams: GD2 to PND10	Oral (diet)	Phthalate mix doses[1]: 200 – 1000 μg/kg bw/d	F1 offspring: Postnatal: PND0, PND5 and PND10	• Cortical neurogenesis (PND5): lower density of BrdU+ cells for all doses.<br>• Apoptosis in the cortex (PND0, PND5 and PND10): higher density of TUNEL+ cells for Mix-5 at PND10. No effect on the density of TUNEL+ cells for PND0 and PND5 at all doses.	[72]

	C57Bl/6 mouse	F0 adult dams: GD13 to PND15	Oral (diet)	DBP: 50 mg/kg/d	F1 offspring: Postnatal: PND15	• Gene expression and protein amounts in the cortex: higher mRNA and protein levels of D2R. Higher mRNA levels of forkhead box, G-protein coupled receptor 88 and 149, PDE 10a, proenkephalin, regulator of G-protein signaling 9. Lower mRNA levels of transthyretin and Inactive X specific transcripts.<br>• Number of cortical neurons: higher number of Nissl-stained neurons.	[69]

[1] Composition of the mixture in and [9]: 35% DEP, 21% DEHP, 15% DBP, 15% DiNP, 8% DiBP, 5% BBP.

Table S3. Details on the behavioral tests used.

**Behavioral tests**	**Reference**
The lordosis quotient was defined as the number of lordosis postures / 10 male mounts x 100.	[51], [52], [53]
The lordosis quotient was defined as the number of lordosis postures / number of male mounts for 20 min x 100.	[4]
At PND1, litters were culled to 8 pups [67], 10 pups [60],or 6 pups [69] with an equal sex-ratio.	[60], [67], [69]
Pup retrieval test: the test consisted in moving all the 8 pups to each corner of the cage, with one male and one female for each corner.	[67]
Pup retrieval test: the test consisted in moving 3 of all the pups to the opposite side of the nest.	[68], [70]
Stressed condition consisted in the elevated-plus-maze.	[73]
Stressed condition consisted in a 15 min restraint.	[78]
Fear conditioning: the test consisted in a training session (three 30s tones with a 2s footshock, followed by a 2 min interval). The test day 24 h later: 30s tones followed by a 2 min interval.	[82]
Morris water maze: the test consisted in a 4-day learning period. The 5th day, the platform was removed (test session).	[80]
Morris water maze: the test consisted in a 5-day learning period. The day after, the platform was removed (test session). This was followed by a visible platform session.	[81]
Morris water maze: the test consisted in 5-day learning period. 4 weeks later, animals were tested for two days. The day after, the platform position was changed to test reversal and new learning.	[68]
Attentional set-shift test: the test consisted in 11- day habituation and pretraining in the plus maze. On day 12 (set 1), animals were trained to enter a specific arm depending on color, with food as a reward. They had to choose the correct arm 8 consequent times. On day 13 (set 2), they were trained to enter a specific arm depending on texture, with food as a reward. Animals had to complete 80 trials, independently of their choice. A perseverative error was entering an incorrect arm according to the rule from set 1, whereas an omission error was an incorrect choice that does not follow the rewarded stimuli from either set.	[84]
Novel object recognition: the test consisted in 5 min habituation to 2 identical objects. 2 h later, animals were exposed to familiar versus novel object during 5 min.	[83]
Morris water maze: the test consisted in a 6-day learning period. The 7th day (test session), the platform was removed.	[76]
Morris water maze: the test consisted in a 4-day learning period. The 5th day, the platform was removed.	[79]
Attentional set-shift test: the test consisted in 11-day habituation and pretraining days in the plus maze. On day 12 (set 1), animals were trained to enter a specific arm depending on color, with food as a reward. Animals had to choose the correct arm 8 consequent times. On day 13 (set 2), they were trained to enter a specific arm depending on texture, with food as a reward. Animals had to complete 80 trials, independently of their choice. A perseverative error was entering an incorrect arm according to the rule from set 1, omission error was an incorrect choice that did not follow the rewarded stimuli.	[72]
Morris water maze: the test consisted in 4-day learning period. The 5th day, the platform was removed.	[92]
